# The Effect of Amyloid‐Beta on Empathy‐like Behavior in Rats

**DOI:** 10.1002/alz.088658

**Published:** 2025-01-03

**Authors:** Zahra Rostami, Hadi Choubdar Parvin, Fariba Khodagholi, Mitra Ansari

**Affiliations:** ^1^ Shahid beheshti University of Medical Science, Tehran, Tehran Iran (Islamic Republic of); ^2^ Ahvaz Jundishapur University of Medical Sciences, Ahvaz, Ahvaz Iran (Islamic Republic of)

## Abstract

**Background:**

Empathy is a complex behavior enabling individuals to recognize and sense the emotional situation of others. Empathy requires cognitive, emotional, and learning abilities to understand and react to the suffering of others. The current study evaluates the effect of Amyloid‐Beta (Aβ), an aggregated peptide involved in Alzheimer’s disease on empathy‐like behavior.

**Method:**

Aβ was injected bilaterally in the hippocampal tissue of 14 Wistar rats. Aβ‐injected rats were compared to the control group during an empathic behavioral task for 12 days. Empathy was evaluated using the helping behavior of the subject toward his soaking cage‐mate rat. Opening time latencies with or without soaker response were considered as empathy indicators, and their change in two groups was modeled with a linear mixed‐effect model.

**Result:**

Results of the model demonstrated that opening time latency with soaker response, as the major empathy indicator, had decreased in the Aβ group (p‐value = 0.002). Furthermore, the opening time latency without soaker response, the indicator of the helper rat’s solid action independent from social interaction, showed alteration in the Aβ group, the same as the opening time latency with soaker response, which was significantly decreased compared to the control animals (p‐value<0.001).

**Conclusion:**

This result provides good evidence for the disruption of empathy due to a cognitive defect in helper rats’ emotional cue perception during Aβneurotoxicity. Together, these findings help to understand the impact of neurodegenerative disorders on empathic behavior and decode the altered components of empathy in the course of the disease.

